# Transactions of the Indiana State Medical Society

**Published:** 1861-09

**Authors:** 


—THE subject of medical education occupied a large share of the *Transactions of the Indiana State Medical Society* at its annual meeting in May last, at Indianapolis. The address of the President—Dr. B. S. WOODWORTH, of Ft. Wayne—touching the effects of a higher system of medical education and kindred topics, is an out-spoken, sensible effort, abounding in what country folks call "horse-sense"—calling a quack a quack, without indulging in any oily euphuisms, with which to soothe the tender sensibilities of men who defile their Alma Mater and disgrace their cloth by charlatanry and trickery. The committee on the subject of medical education—JAS. F. HIBBARD, M. D., chairman—present an elaborate plan for the remodeling of the present system.

